# Rapid Detection of *mecA* and *femA* Genes by Loop-Mediated Isothermal Amplification in a Microfluidic System for Discrimination of Different Staphylococcal Species and Prediction of Methicillin Resistance

**DOI:** 10.3389/fmicb.2020.01487

**Published:** 2020-07-09

**Authors:** Xiangrui Meng, Guohao Zhang, Bo Sun, Shujun Liu, Yadong Wang, Ming Gao, Yubo Fan, Guojun Zhang, Guangzhi Shi, Xixiong Kang

**Affiliations:** ^1^Laboratory Diagnosis Center, Beijing Tiantan Hospital, Capital Medical University, Beijing, China; ^2^Beijing Engineering Research Center of Immunological Reagents and Clinical Research, Beijing, China; ^3^Intensive Care Unit, Beijing Tiantan Hospital, Capital Medical University, Beijing, China; ^4^Beijing Baicare Biotechnology Co., Ltd., Beijing, China; ^5^School of Biological Science and Medical Engineering, Beihang University, Beijing, China; ^6^School of Medical Science and Engineering, Beihang University, Beijing, China; ^7^Beijing Advanced Innovation Center for Biomedical Engineering, Beihang University, Beijing, China

**Keywords:** microfluidics, staphylococcal infection, loop-mediated isothermal amplification, identification, methicillin resistance

## Abstract

Staphylococcal infection is one of the most pressing problems in modern medicine due to the increasing antibiotic resistance with the overuse of antibiotics. Conventional methods for identification and antimicrobial susceptibility testing (AST) generally take 3–7 days and require skilled technicians. In this study, a microfluidic device based on loop-mediated isothermal amplification (LAMP) was developed, which could discriminate *Staphylococcus aureus*, *Staphylococcus epidermidis*, *Staphylococcus haemolyticus*, and *Staphylococcus hominis* and predict their methicillin resistance by targeting the *mecA* and *femA* genes within 70 min including the hands-on time. Multiplex and real-time detection was achieved in a closed system without aerosol contamination. The limits of detection (LODs) for *S. aureus*, *S. epidermidis*, *S. hominis*, and methicillin-resistant *S. aureus* (MRSA) were 20 CFU/reaction, while that for *S. haemolyticus* was 200 CFU/reaction. A total of 102 positive cultures of cerebrospinal fluid (CSF) were also tested, and the results were in good agreement with those from conventional methods. Furthermore, mixed cultures were readily identified by our method. The portable and integrated device is rapid, accurate, and easy to use, which can provide information for prompt institution of proper antimicrobial therapy and has great potential for clinical applications, especially in resource-limited settings.

## Introduction

As a group of Gram-positive bacteria, *Staphylococcus* strains are responsible for a broad range of clinical infections. Based on their ability to produce coagulase, *Staphylococcus* species can be divided into two groups: coagulase-positive staphylococci (CoPS) and coagulase-negative staphylococci (CoNS). *Staphylococcus aureus*, one major representative of the first group, is a leading cause of infections ranging from mild skin infections to life-threatening diseases such as pneumonia, postsurgical infections, and bacteremia (van de Beek et al., 2010; Holland et al., 2014; Tong et al., 2015a). Although the introduction of methicillin and other semisynthetic penicillins such as oxacillin has improved the treatment outcome of patients with staphylococcal infections, the prevalence of methicillin-resistant *S. aureus* (MRSA) has been increasing since the first report in 1960s (Stefani et al., 2012; Lakhundi and Zhang, 2018), which limits the therapeutic options and thus compromises the outcome (Haddadin et al., 2002). Coagulase-negative staphylococci, on the other hand, are often found on human skin and mucous membranes with low virulence. Therefore, they have long been regarded as harmless skin commensals and dismissed as culture contaminants. However, in recent years, their potential role as pathogens has been recognized with the widespread use of broad-spectrum antibiotics and the increasing frequency of invasive medical procedures (von Eiff et al., 2002; Becker et al., 2014), and their incidence has been increasing and become even greater than that of *S. aureus* in nosocomial infections, with *Staphylococcus epidermidis*, *Staphylococcus haemolyticus*, and *Staphylococcus hominis* being the three most common species (Spanu et al., 2003; Tian et al., 2015). Worse still, since most infections caused by CoNS are nosocomial, they have shown higher resistance rates to antibiotics than *S. aureus* (Najar-Peerayeh et al., 2014) and can also serve as reservoirs of resistance genes that may be transferred to other pathogens (Wielders et al., 2001). In these settings, early diagnosis and prompt institution of appropriate antimicrobial therapy without overuse of antibiotics are of great significance, where rapid identification of staphylococci and detection of methicillin resistance are essential.

Nosocomial bacterial meningitis is one of the most dreaded infections mainly caused by staphylococci, either CoNS or *S. aureus* (van de Beek et al., 2010; Tian et al., 2015). The conventional methodology used for diagnosis involves several consecutive steps including enrichment culture of cerebrospinal fluid (CSF), isolation of colonies on solid media, identification by biochemical tests or matrix-assisted laser desorption ionization-time of flight mass spectrometry (MALDI-TOF MS), and antimicrobial susceptibility testing (AST), which requires a lengthy time span of 3–7 days and skilled technicians. To achieve rapid identification of pathogens and detection of antibiotic resistance, molecular techniques such as polymerase chain reaction (PCR), multiplex PCR, and loop-mediated isothermal amplification (LAMP) have been investigated during the last decades (Notomi et al., 2000; Mason et al., 2001). Due to ease of performance and less time consumption, LAMP assays have been integrated into microfluidic systems to work in an automated manner for on-site detection of pathogens in recent years (Jiang et al., 2016b; Lee et al., 2016; Ma et al., 2019; Zhang et al., 2019). However, most of these studies focused only on *S. aureus* and its methicillin susceptibility, and end-point detection was usually performed because of its convenience although it might lead to misinterpretation of the results. Real-time detection, on the other hand, can provide adequate information of the amplification processes and help to improve the accuracy. Few studies have integrated LAMP reaction and real-time detection in a portable device for simultaneous identification of different staphylococcal species and methicillin susceptibility testing (Zheng et al., 2020), which is in urgent need for patients to receive proper therapy as soon as possible.

Studies have demonstrated that methicillin-resistant strains produced a low-affinity penicillin-binding protein (PBP2a or PBP2’) encoded by the *mecA* gene, which conferred methicillin resistance in staphylococci (Ubukata et al., 1989; Chambers, 1997). Since *mecA* was highly conserved and widely disseminated among multiple staphylococcal species, it was hypothesized that it could be carried on a mobile genetic element having the capacity to transfer among staphylococci, which was subsequently identified and designated as staphylococcal cassette chromosome *mec* (SCC*mec*) (Ito et al., 1999; Katayama et al., 2000; Lakhundi and Zhang, 2018). There are three basic structural elements in SCC*mec*: the *mec* complex, the *ccr* complex, and the joining regions. The *mec* complex, which comprises the *mec* gene, its regulator genes and the associated insertion sequences, has been categorized into five classes (A–E) based on differences in insertion sequences and regulatory elements upstream and downstream of the *mec* gene. The *ccr* complex, which contains genes that mediate the insertion and excision of the SCC*mec*, has been given type numbers 1–9 based on different combinations of *ccr* allotypes. According to the combination of different classes of the *mec* complex and types of the *ccr* complex, 14 types of SCC*mec* elements (I–XIV) have been reported so far (Baig et al., 2018; Lakhundi and Zhang, 2018; Urushibara et al., 2020). Although SCC*mec* elements are highly diverse in their structural organization and genetic content, the *mecA* determinant is highly conserved among methicillin-resistant staphylococci and thus is a useful marker of methicillin resistance (Francois et al., 2007). In addition to *mecA*, other chromosomal factors such as *femA* are also associated with the expression of methicillin resistance (Berger-Bächi et al., 1989). The *femA* gene, which encodes a protein precursor involved in peptidoglycan biosynthesis and has been used as a molecular marker for the identification of *S. aureus* (Jukes et al., 2010), is also phylogenetically conserved with a divergence of ∼20% among the staphylococci (Vannuffel et al., 1999). Therefore, methicillin resistance can be predicted by LAMP assays targeting the *mecA* gene, while differential identification of staphylococcal species can be realized by using primers complementary to the interspecies variable regions of the *femA* genes (Jukes et al., 2010; Xu et al., 2012).

The aim of this study was to develop a LAMP-based microfluidic device that could discriminate *S. aureus*, *S. epidermidis*, *S. haemolyticus*, and *S. hominis*, four most clinically common staphylococcal species causing bloodstream infections and nosocomial bacterial meningitis (Spanu et al., 2003; Tian et al., 2015), and predict their methicillin resistance by targeting the *mecA* and *femA* genes. First, the system consisted of the microfluidic chip, and the instrument was established and tested. Then, the limits of detection (LODs) and specificities of the assays were determined and validated. Finally, 102 CSF cultures that were growth positive for *S. aureus*, *S. epidermidis*, *S. haemolyticus*, or *S. hominis* detected by conventional methods were tested to further evaluate the performance of our system.

## Materials and Methods

### Materials and Reagents

All the reagents were from commercial sources and used without further purification. All the solutions were prepared using water from a Synergy UV Water Purification System (Millipore, Billerica, MA, United States). The kits for bacterial DNA extraction were from Biocare (Tianjin) Biotechnology Co., Ltd. (Tianjin, China). Syringe filters with pore size of 5 μM were from Taoyuan Medical and Chemical Instrument Factory (Jiaxing, China). The LAMP kits containing Bst 2.0 WarmStart DNA Polymerase, 10× isothermal amplification buffer, deoxyribonucleotide triphosphate (dNTP) mix, and MgSO_4_ were from New England BioLabs (Beverly, MA, United States). The GoTaq Probe qPCR Master Mix was from Promega Corporation (Madison, WI, United States). The primers, probe, and the positive control template were synthesized by Sangon Biotech Co., Ltd. (Shanghai, China). Bovine serum albumin (BSA) and betaine were from Sigma-Aldrich (St. Louis, MO, United States). EvaGreen was from Biotium, Inc. (Hayward, CA, United States). The real-time PCR system (LightCycler 480) was from Roche Diagnostics (Mannheim, Germany).

Reference strains involved in this study to evaluate the specificities of the LAMP assays were as follows: *S. aureus* (CMCC 26003), *S. epidermidis* (ATCC 49134), *S. haemolyticus* (CGMCC 1.10528), *S. hominis* (ATCC 27844), *Staphylococcus warneri* (ATCC 17917), *Staphylococcus capitis* (ATCC 35661), *Staphylococcus cohnii* (ATCC 29974), *S. aureus* (ATCC 43300), *Staphylococcus argenteus* (CGMCC 1.802), *Staphylococcus lugdunensis* (ATCC 700328), *Staphylococcus saprophyticus* (ATCC 49907), *Staphylococcus auricularis* (ATCC 33753), *Staphylococcus chromogenes* (ATCC 43764), *Staphylococcus pettenkoferi* (DSM 19554), *Staphylococcus pseudintermedius* (ATCC 51874), *Escherichia coli* (CMCC 44102), *Klebsiella pneumoniae* (CMCC 46117), *Pseudomonas aeruginosa* (CMCC 10211), *Acinetobacter baumannii* (CICC 22933), *Enterococcus faecium* (ATCC 35667), *Enterobacter cloacae* (CMCC 43501), *Stenotrophomonas maltophilia* (CICC 22935), *Streptococcus pneumoniae* (CMCC 31001), *Acinetobacter calcoaceticus* (ATCC 23055), *Proteus mirabilis* (ATCC 7002), and *Streptococcus pyogenes* (ATCC 19615). For the LOD tests, several clinically isolated strains of *S. aureus*, *S. epidermidis*, *S. haemolyticus*, and *S. hominis* were also used. All the clinically isolated strains were preliminarily identified by the matrix-assisted laser desorption ionization-time of flight mass spectrometry (MALDI-TOF MS) system.

The automated culture system (BacT/ALERT 3D), the MALDI-TOF MS system (VITEK MS), and the antimicrobial susceptibility testing system (VITEK-2 Compact) were all from bioMérieux, Inc. (Marcy l’Etoile, France). The blood agar plates were from Thermo-Fisher Biochemical Products (Beijing) Co., Ltd. (Beijing, China).

### Primer Design

The sequences of the *mecA* and *femA* genes were acquired from the National Center for Biotechnology (NCBI) GenBank website^1^ under accession numbers KC243783.1, AP020316.1, U23713.1, U23711.1, and Y12874.1, respectively, and were analyzed using the Basic Local Alignment Search Tool^2^. The LAMP primers were designed using PrimerExplorer V5^3^, and those targeting the conserved regions of the *mecA* gene and the interspecies variable regions of the *femA* genes were selected. The nucleotide sequences of the primers are listed in Table 1. As shown in Table 1, four to six primers were involved in each LAMP assay including two outer primers (F3 and B3), two inner primers (FIP and BIP), and loop primers (LF and/or LB). The loop primers were not necessarily required but could accelerate the LAMP reaction. The PCR primers and probe for the *mecA* gene were designed using Primer Express (Applied Biosystems, Foster City, CA, United States), and the sequences are listed in Supplementary Table S1.

**TABLE 1:** The sequences of loop-mediated isothermal amplification (LAMP) primers.

Target gene	Accession number	Primer name	Sequence	Position
*MecA* gene	KC243783.1	F3	TTATGGCTCAGGTACTGCT	1029–1047
		B3	TTTTGTTATTTAACCCAATCATTGC	1228–1252
		FIP	ATTCTTCGTTACTCATGCCATACAT-GTGAATTATTAGCACTTGTAAGCAC	1064–1088, 1114–1138
		BIP	AACCGAAGATAAAAAAGAACCTCTG-AATATTTTTTGAGTTGAACCTGGTG	1149–1173, 1199–1223
		LF	AATGGATAGACGTCATATGAAGGT	1089–1112
		LB	CTCAACAAGTTCCAGATTACAACTT	1174–1198
*FemA-SAU* gene (specific for *S. aureus*)	AP020316.1	F3	GTGCCTTTACAGATAGCATG	35–54
		B3	GAAAAAGTGTACGAGTTCTTGA	246–267
		FIP	GTTTCATAACCTTCAGCAAGCTTT-CCATACAGTCATTTCACGCA	55–74, 96–119
		BIP	GAGGTCATTGCAGCTTGCTTACT-TCGATCACTGGACCGCG	151–173, 220–236
		LF	AACTCATAGTGGCCAACA	78–95
		LB	GTACCTGTTATGAAAGTGTTCA	181–202
*FemA-SEP* gene (specific for *S. epidermidis*)	U23713.1	F3	GCAATGAATTACCCATCTCTG	938–958
		B3	AAAGTCACCACTAATACCATAG	1104–1125
		FIP	CGATTTGAAGTTCCACCAGCG-CTGGCTTCTTTATAATTAATCCGT	959–982, 999–1019
		BIP	TATCGCCATTTTGCAGGGAGC-TATACCGATTAATACCATGTTCA	1021–1041, 1077–1099
		LB	ATGCGGTTCAATGGAAGATGAT	1043–1064
*FemA-SHA* gene (specific for *S. haemolyticus*)	U23711.1	F3	GCCATATAGTCATTTCACACAA	54–75
		B3	GAAAAAGTGAACAAGCTCTCT	247–267
		FIP	GGCTGCAATAACCTCATTATCTTT-CTATGAGATGAAAGGTGCAAAT	87–108, 142–165
		BIP	TGCATGTTGACAGCAGTACCAG-TCATAATCAATTACAGGTCCTC	166–187, 221–242
		LF	ACCAACTAAGTGAGTTTCTGTTTT	109–132
*FemA-SHO* gene (specific for *S. hominis*)	Y12874.1	F3	GCTACAGAATTTGGCGATT	22–40
		B3	AAAAGTGAACGAGTTCTTTGT	245–265
		FIP	AGTTTCAGTTTTCTCAGCAACTTTT-GCCATATAGCCATTTTACACA	54–74, 96–120
		BIP	GAAGTCATTGCTGCTTGTATGCTA-AATGACTGGACCACGATT	151–174, 217–234
		LB	ACTGCTGTACCCGTTATGAAAAT	175–197

### DNA Extraction

The crude DNA was extracted according to the manufacturer’s instructions. Briefly, 200 μl bacterial suspension was centrifuged at a speed of 12,000 rpm for 2 min, and the supernatant was discarded. Then, the pellets were resuspended in 200 μl extracting solution containing 10 mM Tris–HCl, 1 mM ethylenediaminetetraacetic acid (EDTA), and protectants of nucleic acids and transferred into the lysis tube containing glass beads. After vortexing for 5 min and subsequent incubation at 100°C for 5 min, the tube was centrifuged briefly to obtain the supernatant. For positive cultures of CSF, the samples were filtrated through syringe filters with pore size of 5 μM to remove activated carbon particles in the culture broth while retaining the bacteria to be tested prior to DNA extraction. All the above steps for nucleic acid extraction took ∼15 min to complete.

### LAMP and PCR Reaction

For off-chip LAMP assays, the 10-μl reaction mixture contained 1× isothermal amplification buffer, 1.4 mM each dNTP, 6 mM MgSO_4_, 0.2 μM each outer primer (F3 and B3), 1.6 μM each inner primer (FIP and BIP), 0.8 μM each loop primer (LF and LB), 3.2 U Bst 2.0 WarmStart DNA Polymerase, 0.5 mg/ml BSA, 0.8 M betaine, 0.6× EvaGreen, and 2 μl extracted DNA template. Water was used as no template control (NTC). Amplification and melting curve analysis were performed in the LightCycler 480 system as follows: 65°C for 50 cycles of 1 min, followed by 95°C for 1 min with a ramp rate of 4.4°C/s, 55°C for 1 min with a ramp rate of 2.2°C/s, and a slow incremental increase to 95°C with a ramp rate of 0.04°C/s. For on-chip LAMP assays, the reaction mixture was similarly prepared except that the primers were preloaded in the reaction chambers of the microfluidic chip.

For PCR assays of the *mecA* gene, the 20 μl reaction mixture contained 1× GoTaq Probe qPCR Master Mix, 0.3 μM forward and reverse primers, 0.15 μM probe, and 2 μl extracted DNA template. Water was used as no template control (NTC). The assays were performed in the LightCycler 480 system as follows: polymerase activation at 95°C for 5 min, followed by 45 amplification cycles of 95°C for 30 s and 60°C for 30 s. The fluorescence was detected after the annealing-extension step of each cycle.

### Microfluidic Chip and Instrument

**FIGURE 1:**
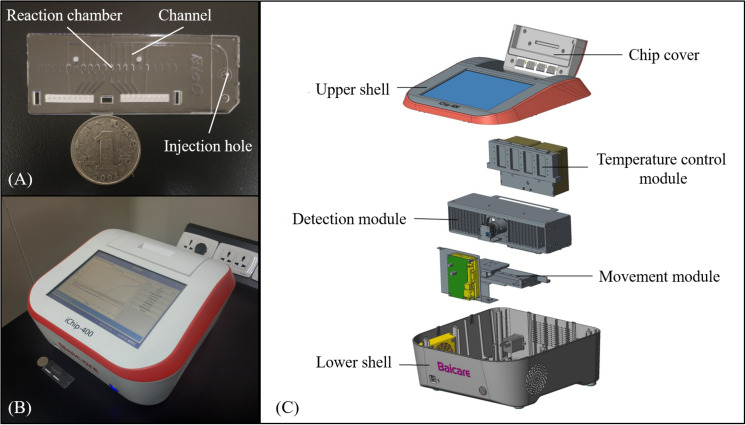
The microfluidic chip and instrument. **(A)** Photograph of the chip that includes reaction chambers connected by channels. **(B)** Photograph of the instrument with results shown on the screen. **(C)** Explosive view of the instrument.

The microfluidic chips employed in this study were designed by Solidworks 2014 software and were injection molded using polycarbonate (PC). As shown in Figure 1A, the chip contained 10 independent reaction chambers with a volume of ∼4 μl, which were connected by long thin channels with a width of 500 μM and a depth of 200 μM. For chip assembly, the injection-molded PC layer was first washed with ethanol and pure water for 2 min each using an ultrasonic cleaning machine and dried at 80°C for 3 h in an oven. Various sets of primers listed in Table 1 were then preloaded and dried at room temperature for 1 h in the corresponding reaction chambers, respectively, as given in Supplementary Table S2. For positive control, the template and primers listed in Supplementary Table S3 were embedded simultaneously in the same chamber, while for negative control, the chamber remained empty. Subsequently, the microfluidic chips were sealed carefully with pressure sensitive tapes and were then stored at 4°C before use.

As shown in Figures 1B,C, a portable instrument was developed with a dimension of 35 × 34 × 18 cm and a weight of 8 kg. The instrument mainly consisted of the temperature control module, the detection module, and the movement module. The temperature control module provided a constant temperature of 65°C for isothermal amplification using a Peltier heater (12706, Fulianjing Electronics Co., Ltd., Qinhuangdao, China). For the detection module, a blue LED (3 W, Wenliang Electronics Co., Ltd., Shenzhen, China) and an optical filter (470 nm ± 15 nm, Jingyi Bodian Optical Technology Co., Ltd., Beijing, China) were used to excite the EvaGreen dye, and the fluorescence emitted from the reaction chambers through an optical filter (520 nm ± 10 nm, Jingyi Bodian Optical Technology Co., Ltd., Beijing, China) was photographed by a high-definition camera (CM3-U3-12S2M, FLIR, Canada) during the LAMP reaction, based on which the intensity was calculated using OpenCV, and the real-time curve was plotted and displayed on the screen. With the aid of the movement module, the instrument could process four microfluidic chips simultaneously in a batch. Details of the instrument are illustrated in Supplementary Figure S1.

### Operation of On-Chip Tests

After the nucleic acids were extracted as described in section “DNA Extraction,” they were mixed with the reaction solution, which contained Bst 2.0 WarmStart DNA Polymerase, dNTP mix, EvaGreen, etc. The mixture (∼70 μl) was then injected into the microfluidic chip using a micropipette, following which the injection hole was sealed by a tape. Finally, the microfluidic chips were inserted into the instrument, which performed the amplification and real-time detection in 50 min. The whole procedure is illustrated in Supplementary Figure S2, which was completed within 70 min including the hands-on time.

### LOD and Specificity Tests

To determine the LODs of the LAMP assays, the bacterial strains were cultured and the concentrations were estimated by plate counting, following which they were serially diluted to 10^4^, 10^3^, 10^2^, 10^1^, and 10^0^ CFU/μl. Then, the crude DNA was extracted and amplified, respectively, as described above. For specificity tests, the experiments were conducted similarly except that the concentrations were all adjusted to 10^5^ CFU/μl.

### Clinical Sample Tests

This study was performed at Beijing Tiantan Hospital, a tertiary hospital in Beijing, and was approved by Beijing Tiantan Hospital’s ethics committee. Informed consent was obtained from the patients or their families. A total of 102 CSF cultures positive for *S. aureus*, *S. epidermidis*, *S. haemolyticus*, or *S. hominis* were collected between May 2019 and October 2019. Identification and antimicrobial susceptibility tests were carried out using standard procedures. After the CSF cultures were detected as positive by the BacT/ALERT 3D system, an aliquot of the culture broth was inoculated onto blood agar and incubated at 37°C in an incubator for 18–24 h. The isolates were then identified by MALDI-TOF MS using the VITEK MS system, and the antimicrobial susceptibility tests were performed using the VITEK-2 Compact system, in which oxacillin was used to determine the methicillin susceptibility. To evaluate the performance of our system, the positive culture broth was also taken out and tested as described in sections “DNA Extraction,” “LAMP and PCR Reaction,” “Microfluidic Chip and Instrument,” and “Operation of On-Chip Tests.”

## Results

### Performance of the Instrument

The performance of our instrument was evaluated according to YY/T 1173-2010 guidelines. For repeatability tests, the fluorescence intensity of the reaction chambers was tested 10 times using 5 different concentrations of fluorescent dye, and the coefficient of variation (CV) was <0.7% as shown in Supplementary Table S4. The precision of our instrument was evaluated by testing the fluorescence intensity of each chamber in one chip, and the CV was <3.2% as shown in Supplementary Table S5. The linearity of fluorescence intensity was also evaluated with the correlation coefficient (*R*^2^) >0.99, as shown in Supplementary Figure S3. Temperature control is another important property of the instrument. The performance of our instrument was shown in Supplementary Tables S6–S8 with mean heating rate higher than 18.2°C/min and mean cooling rate higher than 15.4°C/min, and the channels for chips were maintained at 65 ± 0.3°C.

### Testing of Cross-Contamination

**FIGURE 2:**
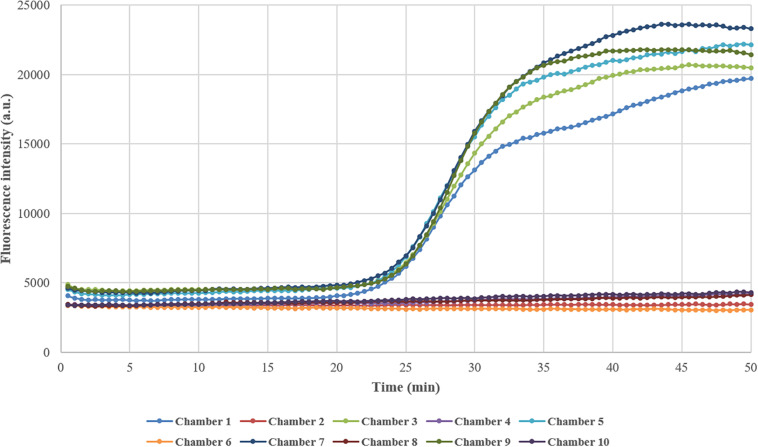
Testing of cross-contamination between adjacent reaction chambers. Chambers in odd numbers were embedded with primers in advance, while chambers in even numbers remained empty. After the reaction mixture containing DNA template and other reagents was loaded into the chip and amplification was performed, the fluorescence intensity in odd-numbered chambers increased greatly while that in even-numbered chambers remained almost unchanged.

In this study, the reaction chambers in the microfluidic chip were connected by channels filled with liquid and were not isolated from each other during DNA amplification, which raised the possibility of cross-contamination between adjacent chambers. To determine whether the reactions interfered with each other, the reaction chambers in odd numbers were embedded with primers for *femA-SHO* gene while the chambers in even numbers remained empty during the preparation of the chip, and the reference strain *S. hominis* ATCC 27844 with a concentration of 10^4^ CFU/μl was used as described above. After the reaction mixture containing the extracted DNA template of *S. hominis* ATCC 27844 and other reagents was injected into the chip, amplification was performed, and the fluorescence was detected real-time by the instrument. As expected, the fluorescence in odd-numbered chambers increased significantly, indicating the presence of gene amplification, while that in even-numbered chambers remained almost unchanged during the process as depicted in Figure 2, which demonstrated that there was no cross-contamination between reaction chambers.

### LODs and Specificities of the LAMP Assays

**FIGURE 3:**
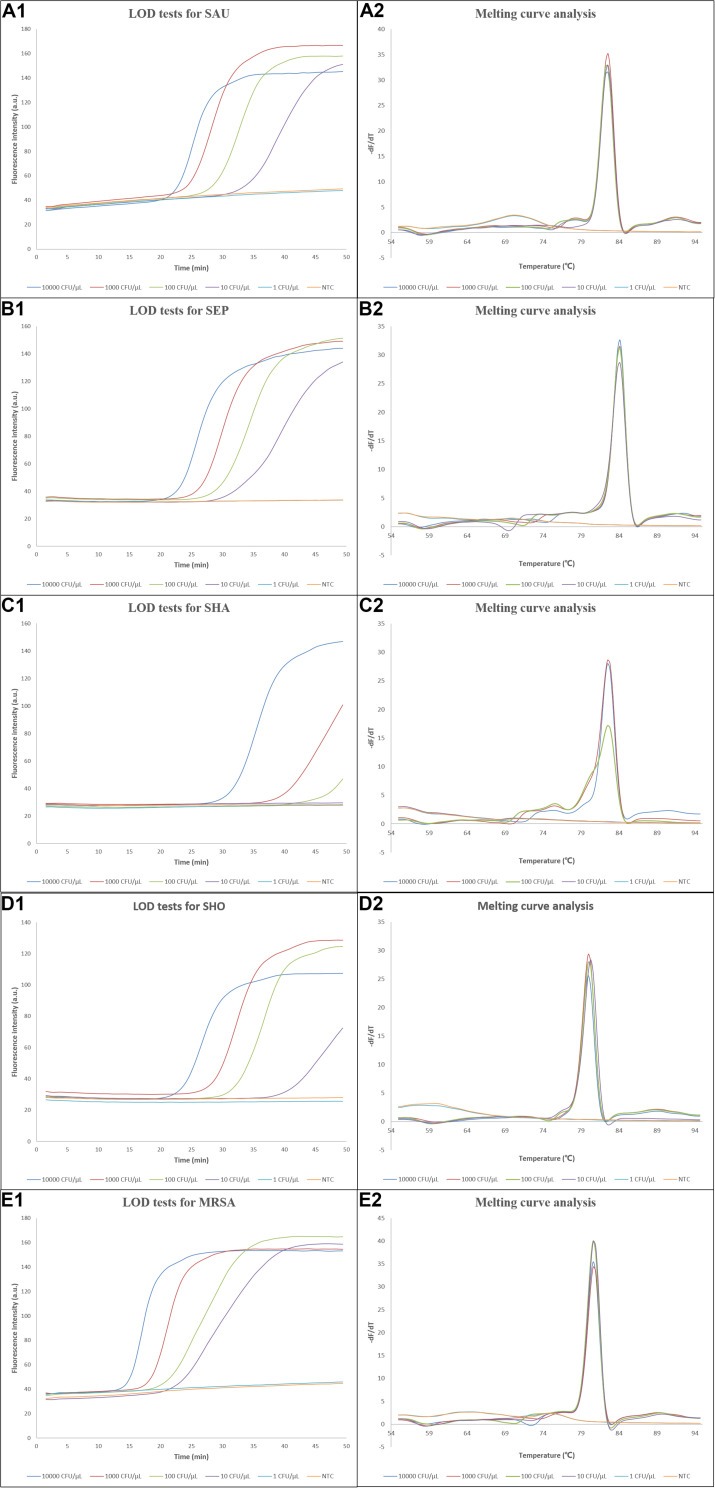
Results of the LOD tests. **(A1–E1)** Amplification curves of the LOD tests for *S. aureus*, *S. epidermidis*, *S. haemolyticus*, *S. hominis*, and methicillin-resistant *S. aureus*, respectively, and **(A2–E2)** the corresponding melting curve analysis. LOD, limit of detection; dF/dT, derivative of the fluorescence/derivative of the temperature; SAU, *Staphylococcus aureus*; SEP, *Staphylococcus epidermidis*; SHA, *Staphylococcus haemolyticus*; SHO, *Staphylococcus hominis*; MRSA, methicillin-resistant *Staphylococcus aureus*; NTC, no template control.

To estimate the LODs of the LAMP assays, the reference strains *S. aureus* (CMCC 26003), *S. epidermidis* (ATCC 49134), *S. haemolyticus* (CGMCC 1.10528), *S. hominis* (ATCC 27844), and methicillin-resistant *S. aureus* (ATCC 43300) were used in the tests, and water was used as no template control (NTC). As shown in Figure 3, the DNAs extracted from *S. aureus*, *S. epidermidis*, *S. hominis*, and MRSA with concentrations ranging from 10,000 to 10 CFU/μl and *S. haemolyticus* from 10,000 to 100 CFU/μl were successfully amplified, which were also verified by the corresponding melting curve analysis, and the initiation time of amplification gradually increased with decreasing concentrations of reference strains. Owing to the use of 2 μl crude DNA extract in each assay, the LODs for *S. aureus*, *S. epidermidis*, *S. hominis*, and MRSA reached ∼20 CFU/reaction, while that of S. haemolyticus was 200 CFU/reaction. Several clinically isolated strains were also tested, which demonstrated similar or better LODs as shown in Supplementary Figures S4–S8. For on-chip LOD tests, the results were the same as those in tubes, which were comparable to other studies (Xu et al., 2012; Jiang et al., 2016a; Huang et al., 2017; Trinh et al., 2019) and also demonstrated that our system had a similar performance compared to bench-top equipment.

**FIGURE 4:**
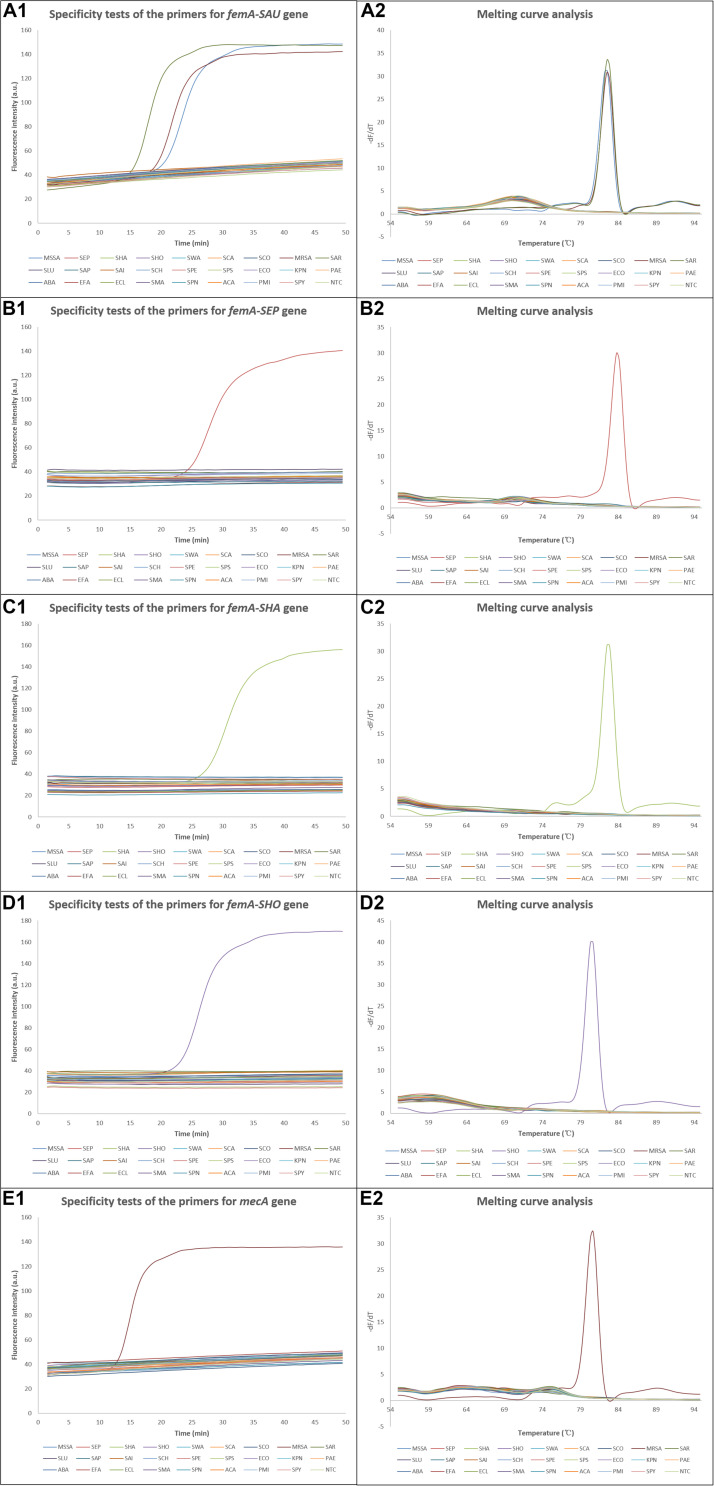
Results of the specificity tests. **(A1–E1)** Amplification curves of the specificity tests for *S. aureus*, *S. epidermidis*, *S. haemolyticus*, *S. hominis*, and methicillin-resistant *S. aureus*, respectively, and **(A2–E2)** the corresponding melting curve analysis. dF/dT, derivative of the fluorescence/derivative of the temperature; SAU, *Staphylococcus aureus*; MSSA, methicillin-susceptible *Staphylococcus aureus*; SEP, *Staphylococcus epidermidis*; SHA, *Staphylococcus haemolyticus*; SHO, *Staphylococcus hominis*; SWA, *Staphylococcus warneri*; SCA, *Staphylococcus capitis*; SCO, *Staphylococcus cohnii*; MRSA, methicillin-resistant *Staphylococcus aureus*; SAR, *Staphylococcus argenteus*; SLU, *Staphylococcus lugdunensis*; SAP, *Staphylococcus saprophyticus*; SAI, *Staphylococcus auricularis*; SCH, *Staphylococcus chromogenes*; SPE, *Staphylococcus pettenkoferi*; SPS, *Staphylococcus pseudintermedius*; ECO, *Escherichia coli*; KPN, *Klebsiella pneumoniae*; PAE, *Pseudomonas aeruginosa*; ABA, *Acinetobacter baumannii*; EFA, *Enterococcus faecium*; ECL, *Enterobacter cloacae*; SMA, *Stenotrophomonas maltophilia*; SPN, *Streptococcus pneumoniae*; ACA, *Acinetobacter calcoaceticus*; PMI, *Proteus mirabilis*; SPY, *Streptococcus pyogenes*; NTC, no template control.

Since the system was intended for clinical applications, the ability to exclusively identify the four most clinically common staphylococcal species from each other and other clinically common pathogens was essential. The specificities of the LAMP assays were confirmed using DNAs extracted from bacterial strains with a concentration of 10^5^ CFU/μl. As shown in Figure 4, amplification was successful when the corresponding DNA template was added, while the amplification curves were similar to that of NTC when non-corresponding DNA templates were used, which was also verified by the melting curve analysis. The exception was that the template of *S. argenteus* was also amplified by the primers for *femA-SAU* gene, which was discussed later. For on-chip tests, no amplification was observed in reaction chambers except the positive control chamber when using non-corresponding DNA templates, as expected. Thus, these results demonstrated high specificity of the LAMP assays with multiple primers involved in each assay and fine discrimination of different staphylococcal species by targeting the variable regions of the *femA* genes.

### Results of Clinical Sample Tests

To further investigate the performance of our system, a total of 102 positive CSF cultures were tested, and the results were compared with those obtained by conventional methods. As listed in Table 2, our system correctly identified 11 MSSA and 4 MRSA with 11 results positive for *femA-SAU* only and 4 results positive for both *femA-SAU* and *mecA*, respectively. The results were also in good agreement with respect to staphylococcal species identification and methicillin-resistance determination for methicillin-susceptible *S. epidermidis*, methicillin-resistant *S. epidermidis* and methicillin-susceptible *S. haemolyticus* cultures. For methicillin-resistant *S. haemolyticus* cultures, 14 results obtained by our system were positive for both *femA-SHA* and *mecA* as expected, but there was one sample also positive for *femA-SEP*, which indicated the presence of *S. epidermidis*. Likewise, *femA-SEP* was detected in 1 out of 4 methicillin-susceptible *S. hominis* cultures and 6 out of 16 methicillin-resistant *S. hominis* cultures, while *femA-SHA* was detected in 1 methicillin-resistant *S. hominis* cultures, which also indicated that they were mixed cultures. To reidentify these positive cultures, more colonies were picked up after colony formation on solid media for MALDI-TOF MS identification, and the presence of more than one species were then confirmed, which was in consistence with the results obtained by our system. The presence or absence of the *mecA* gene in the samples was also confirmed by standard PCR assays, and the results were identical with those obtained by our system. Notably, one methicillin-susceptible *S. hominis* culture was *mecA*-positive, which meant that it was phenotypic susceptible but genotypic resistant.

**TABLE 2:** Comparison of the results obtained by conventional methods and our system.

Results obtained by conventional methods	Results obtained by our system					Results obtained by PCR
	*FemA-SAU*	*FemA-SEP*	*FemA-SHA*	*FemA-SHO*	*MecA*	*MecA*
Methicillin-susceptible *S. aureus* (11)	11	0	0	0	0	0
Methicillin-resistant *S. aureus* (4)	4	0	0	0	4	4
Methicillin-susceptible *S. epidermidis* (6)	0	6	0	0	0	0
Methicillin-resistant *S. epidermidis* (44)	0	44	0	0	44	44
Methicillin-susceptible *S. haemolyticus* (3)	0	0	3	0	0	0
Methicillin-resistant *S. haemolyticus* (14)	0	1*	14	0	14	14
Methicillin-susceptible *S. hominis* (4)	0	1*	0	4	1**	1**
Methicillin-resistant *S. hominis* (16)	0	6*	1*	16	16	16

*Mixed cultures. **Phenotypic methicillin susceptibility but genotypic methicillin resistance.

## Discussion

Conventional methods for identification and AST involve several consecutive steps, which requires a lengthy time span and skilled technicians. With genomes of common pathogens being sequenced and resistance genes discovered, rapid identification of pathogens and detection of antibiotic resistance were achieved using PCR and multiplex PCR during the last decades (Notomi et al., 2000; Mason et al., 2001). Despite their advantages of high sensitivity and specificity, PCR-based methods rely on multiple temperature controls and thus require sophisticated thermocyclers, which hinders their applications in point-of-care testing (POCT). Since 2000, a novel method called loop-mediated isothermal amplification (LAMP) has been established and developed, which eliminates thermal cycling steps and allows rapid amplification of nucleic acids in <1 h while retaining high sensitivity and specificity owing to the use of *Bst* DNA polymerase with strand displacement activity and four to six primers recognizing six to eight different regions of the target sequence (Notomi et al., 2000). In addition to ease of performance, less time consumption, and cost effectiveness (Minetti et al., 2016), LAMP assays are also less sensitive to inhibitory substances present in biological or clinical samples than PCR (Kaneko et al., 2007; Mori and Notomi, 2009), saving the time and cost required for relatively strict DNA extraction and purification. In some studies, LAMP assays were performed using bacterial culture or colony directly, even without DNA extraction (Yan et al., 2017). Due to these advantages, LAMP assays have been integrated into microfluidic systems for on-site detection of pathogens in recent years (Jiang et al., 2016b; Lee et al., 2016; Ma et al., 2019; Zhang et al., 2019). Compared with in-tube assays, multiplex and real-time detection can be achieved rapidly in a microfluidic chip without trained technicians or well-equipped laboratories, which shows great promise in clinical applications, especially in resource-limited settings.

In this study, we have developed a LAMP-based microfluidic system that can identify *S. aureus*, *S. epidermidis*, *S. haemolyticus*, and *S. hominis* and predict their methicillin resistance by targeting the *mecA* gene and the interspecies variable regions of the *femA* genes. However, *S. argenteus* cannot be excluded using the primers specific for *S. aureus*, although there are several mismatches between the primers and the *femA* gene of *S. argenteus*. *S. argenteus* is a newly identified coagulase-positive staphylococcal species, which has been previously misidentified as *S. aureus* (Tong et al., 2015b). The percentage identity is 95.72% between *S. aureus* and *S. argenteus* (accession number FR821777.2) when the *femA* gene of *S. aureus* is analyzed using BLAST (see text footnote 2), while the values are <81% between *S. aureus* and other staphylococcal species such as *S. hominis* and *S. epidermidis*. Since the divergence of *femA* gene is relatively low between *S. aureus* and *S. argenteus*, detection of other genes that are not shared between the two species or whole genome sequencing may achieve accurate discrimination as suggested in other studies (Hansen et al., 2017). When the *femA* genes of *S. epidermidis*, *S. haemolyticus*, and *S. hominis* are analyzed by excluding the corresponding species in the database, the highest percentage identities are 82.09, 81.12, and 81.20%, respectively, and high specificity was achieved using our method as shown in Figure 4.

As discovered in other studies (Kobayashi et al., 1994), phenotypic-susceptible but genotypic-resistant *S. hominis* was detected. Although *mecA* is the prerequisite of methicillin resistance and *femA* shows more correlation than other *fem* genes with methicillin resistance (Akcam et al., 2009), other factors also influence the expression of methicillin resistance (Jeljaszewicz et al., 2000). However, methicillin resistance of such strains may be induced by β-lactam antibiotics (Tokue et al., 1992) during chemotherapy, making detection of *mecA* still significant for clinical decision making. On the other hand, since novel methicillin resistance determinants such as *mecC* gene, which is 70% identical to the *mecA* gene and located in a new SCC*mec* structure (SCC*mec* XI), have been discovered in recent years (García-Álvarez et al., 2011; Paterson et al., 2014), methicillin-resistant staphylococci carrying these genes will not be detected using our method, although the frequency is low as reported so far and there were no such cases in this study.

*S. aureus*, *S. epidermidis*, *S. haemolyticus*, and *S. hominis*, four most clinically common staphylococcal species, are responsible over 50% in total of nosocomial bacterial meningitis (Tian et al., 2015). Conventional methods for identification and AST involve inoculation of positive cultures onto solid media for colony isolation, colony selection and preparation of bacterial suspension, and manipulation of equipment, which require skilled technicians and usually take 2–3 days. In this study, the LAMP reactions and real-time detection were performed in a closed system with little risk of contamination. Owing to the design of elongated channels, there was no cross-contamination between adjacent reaction chambers in the microfluidic chip, which served as the basis for multiplex detection. LODs of 20 CFU/reaction for *S. aureus*, *S. epidermidis*, *S. hominis*, and MRSA or 200 CFU/reaction for *S. haemolyticus* were achieved using our method. Although the LODs were acceptable for the detection of clinical samples, the analytical performance could possibly be improved further by using a larger volume of sample. In order to evaluate our system, a total of 102 positive CSF cultures were tested, and the results were in good agreement with those from conventional methods. Compared with conventional methods, identification of staphylococcal species and prediction of their methicillin resistance by our method can be accomplished in a portable and integrated system within 70 min including the hands-on time as illustrated in Supplementary Figure S2, saving at least 36–48 h and showing great promise for institution of timely and proper therapy without overuse of antibiotics. It has been reported that faster identification and antimicrobial susceptibility testing resulted in reduction in hospital stay, antibiotic use, and overall costs (Galar et al., 2012). Furthermore, colony morphologies of staphylococcal species are similar, making it difficult for technicians to discriminate them precisely by naked eyes during selection of colonies for MALDI-TOF MS identification. However, mixed cultures were readily identified by multiplex and real-time detection using our method as described above. In addition, more pathogens might be identified simultaneously by introducing more specific primers into the microfluidic chip. Compared with previous study (Zheng et al., 2020), the system proposed in this study can process four microfluidic chips in a batch, making simultaneous detection of four samples possible. With industrial personal computer and touch screen integrated in the portable instrument, the system can work in a compact manner, which is more suitable for in-field detection.

In this study, mixed cultures were readily identified by the multiplex detection in the microfluidic system. However, one limitation of our method is that it cannot distinguish between MSSA and MR-CoNS in mixed cultures, although none of the clinical samples in this or other studies (Jukes et al., 2010; Kilic et al., 2010) were mixed cultures of MSSA and MR-CoNS. Therefore, it is recommended that isolation of colonies on solid media should be done prior to DNA extraction and multiplex detection for reconfirmation in case that *S. aureus*, CoNS, and *mecA* are all detected in one sample simultaneously in clinical practice.

In summary, our method is rapid, accurate, and user-friendly, which provides timely information for prompt institution of proper antimicrobial therapy and exhibits great potential for clinical applications, especially in resource-limited settings.

## Data Availability Statement

The raw data supporting the conclusions of this article will be made available by the authors, without undue reservation, to any qualified researcher.

## Ethics Statement

The studies involving human participants were reviewed and approved by the Beijing Tiantan Hospital’s Ethics Committee. The patients/participants provided their written informed consent to participate in this study.

## Author Contributions

XM performed most of the experiments and wrote the manuscript. GHZ fabricated the microfluidic chips and the instrument. BS and SL assisted with the sample collection and treatment. YW, MG, YF, and GJZ collaborated in the evaluation of the results. GS and XK designed the experiments. All authors contributed to the article and approved the submitted version.

## Conflict of Interest

GHZ was employed by Beijing Baicare Biotechnology Co., Ltd. The remaining authors declare that the research was conducted in the absence of any commercial or financial relationships that could be construed as a potential conflict of interest.

## Footnotes

1 http://www.ncbi.nlm.nih.gov/genbank/

2 http://blast.ncbi.nlm.nih.gov/Blast.cgi

3 http://primerexplorer.jp/lampv5e/index.html
