# Supplementary figures and images for: Rapid Detection of mecA and femA Genes by Loop-Mediated Isothermal Amplification in a Microfluidic System for Discrimination of Different Staphylococcal Species and Prediction of Methicillin Resistance
Source: Front Microbiol. 2020 Jul 9;11:1487. doi: 10.3389/fmicb.2020.01487 (PMC7367217; doi:10.3389/fmicb.2020.01487)

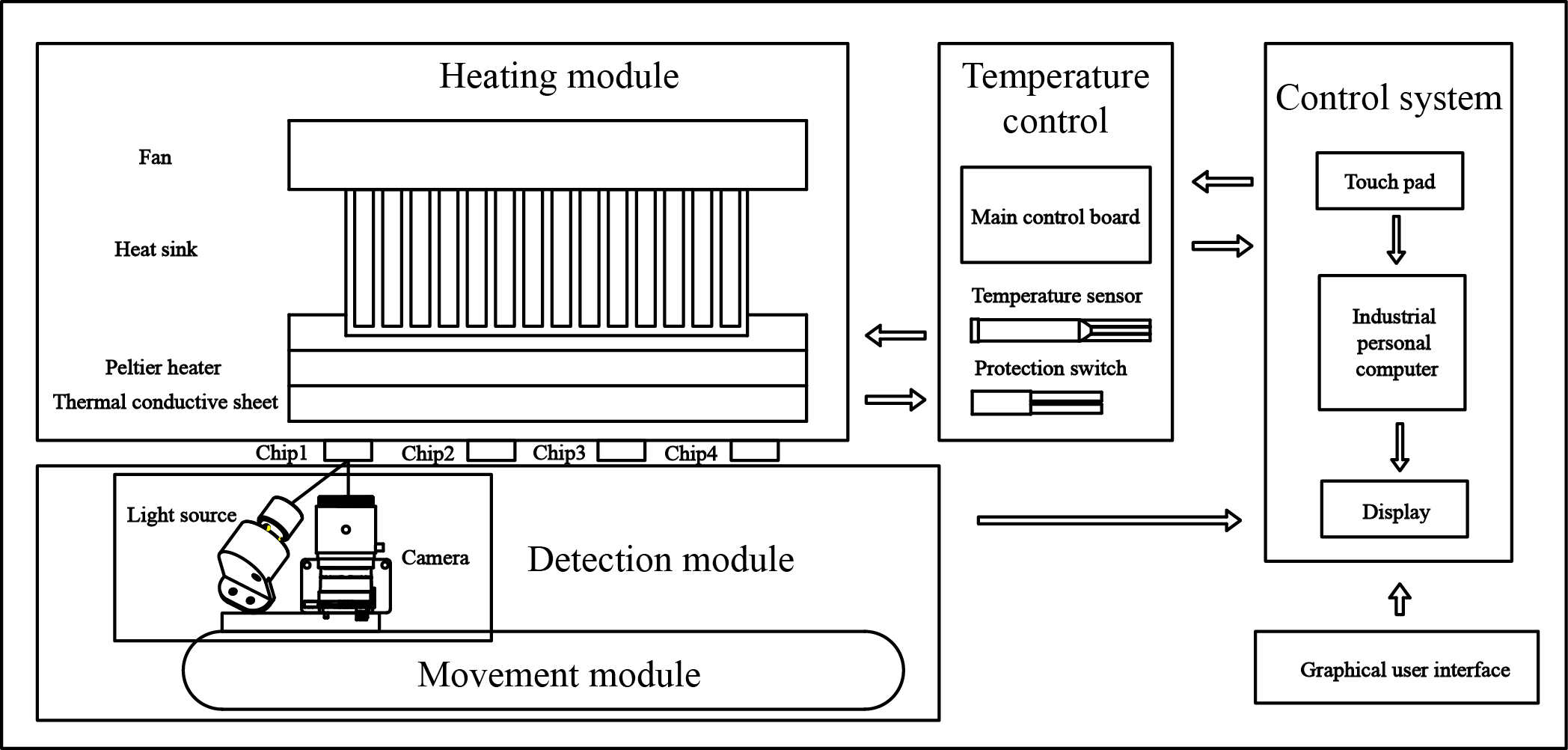

Supplement: Supplementary file 1 [file Image_1.TIF]

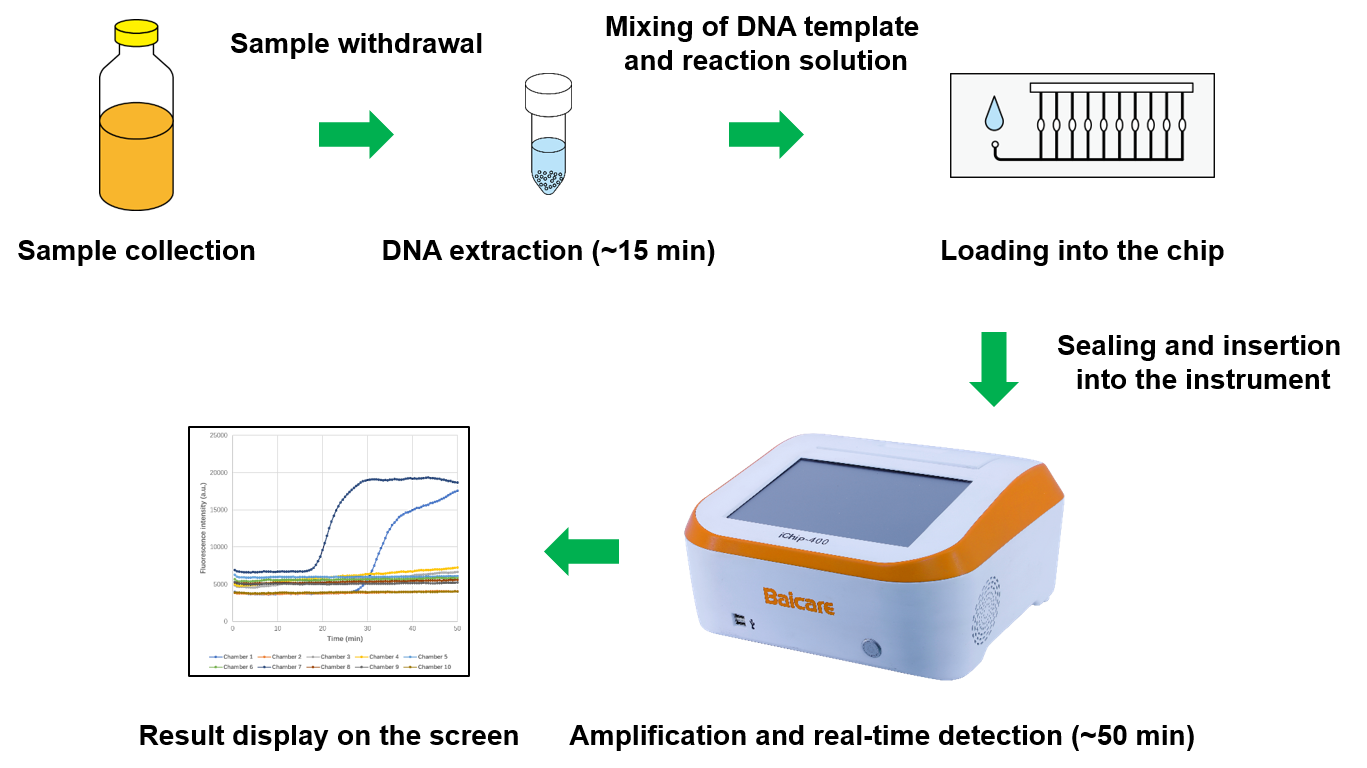

Supplement: Supplementary file 2 [file Image_2.TIF]

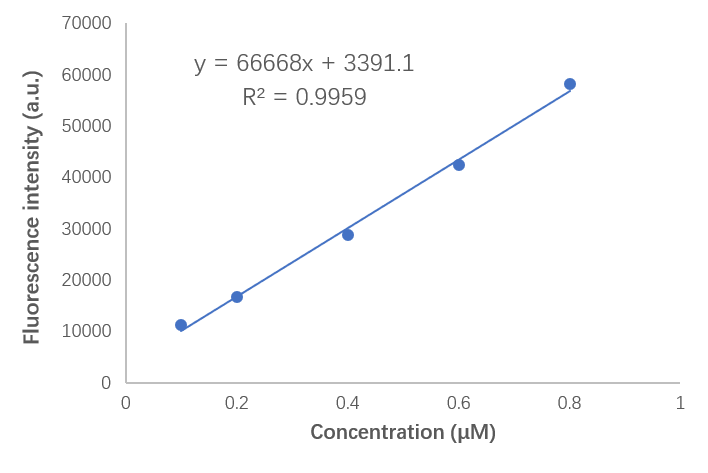

Supplement: Supplementary file 3 [file Image_3.TIF]

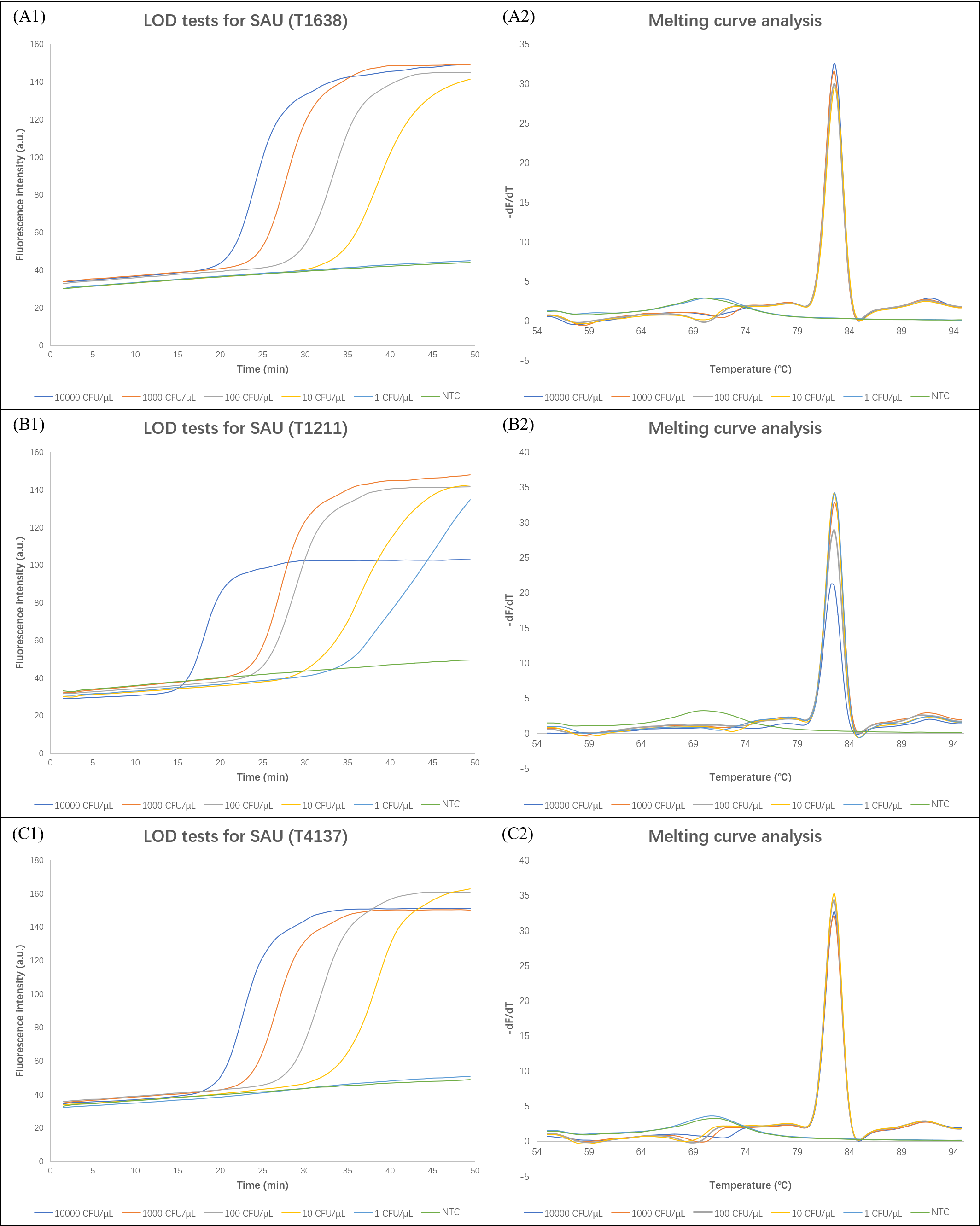

Supplement: Supplementary file 4 [file Image_4.TIF]

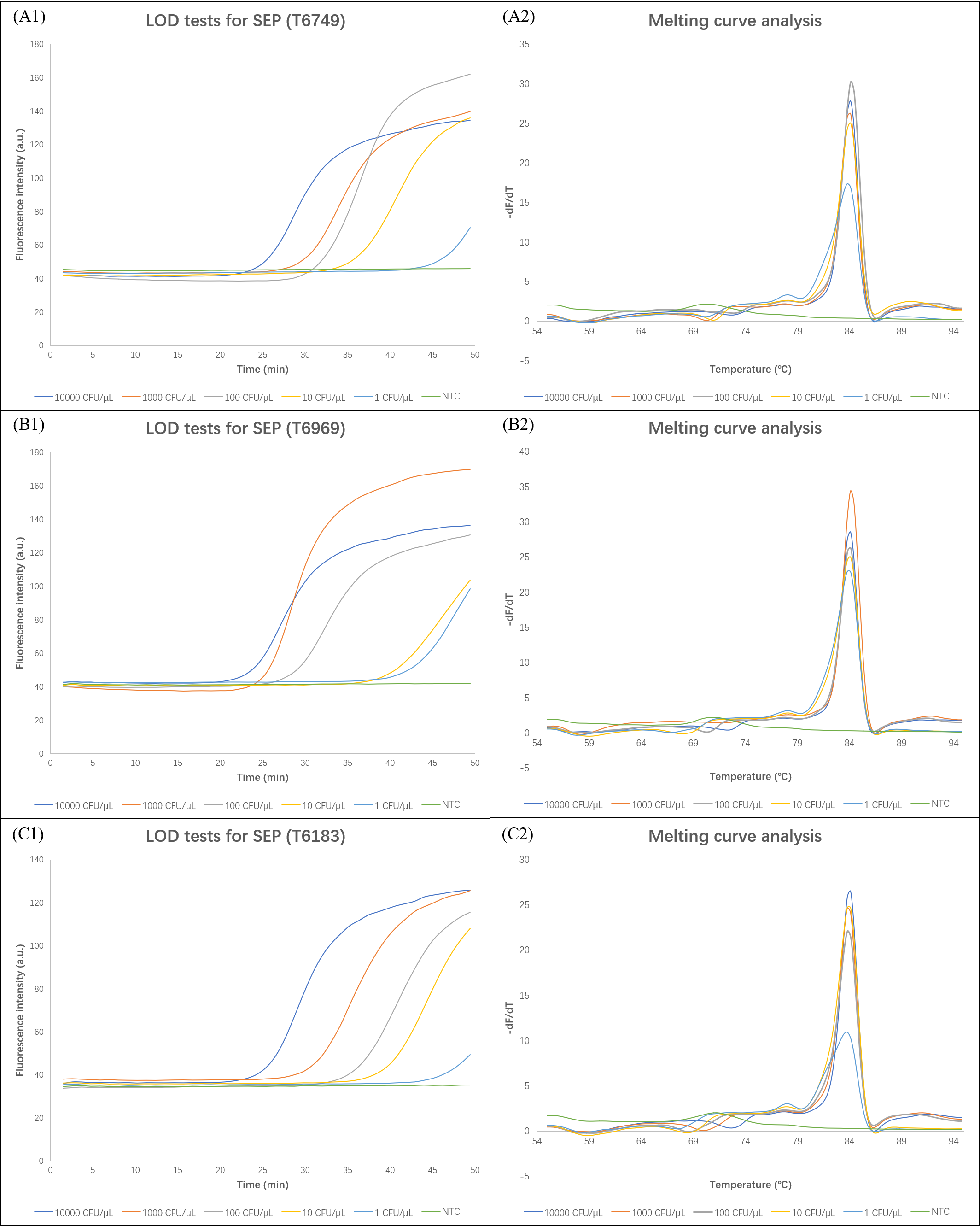

Supplement: Supplementary file 5 [file Image_5.TIF]

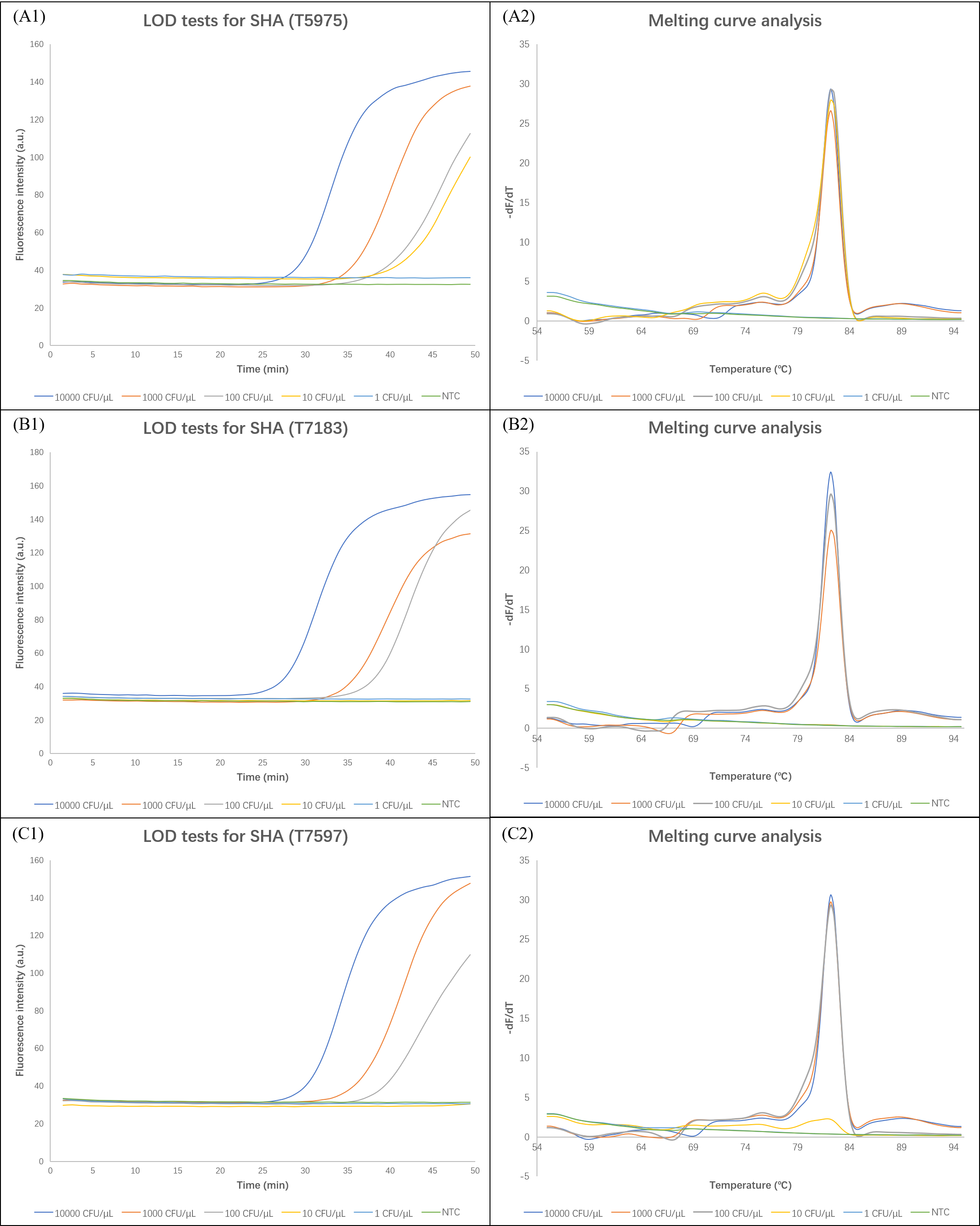

Supplement: Supplementary file 6 [file Image_6.TIF]

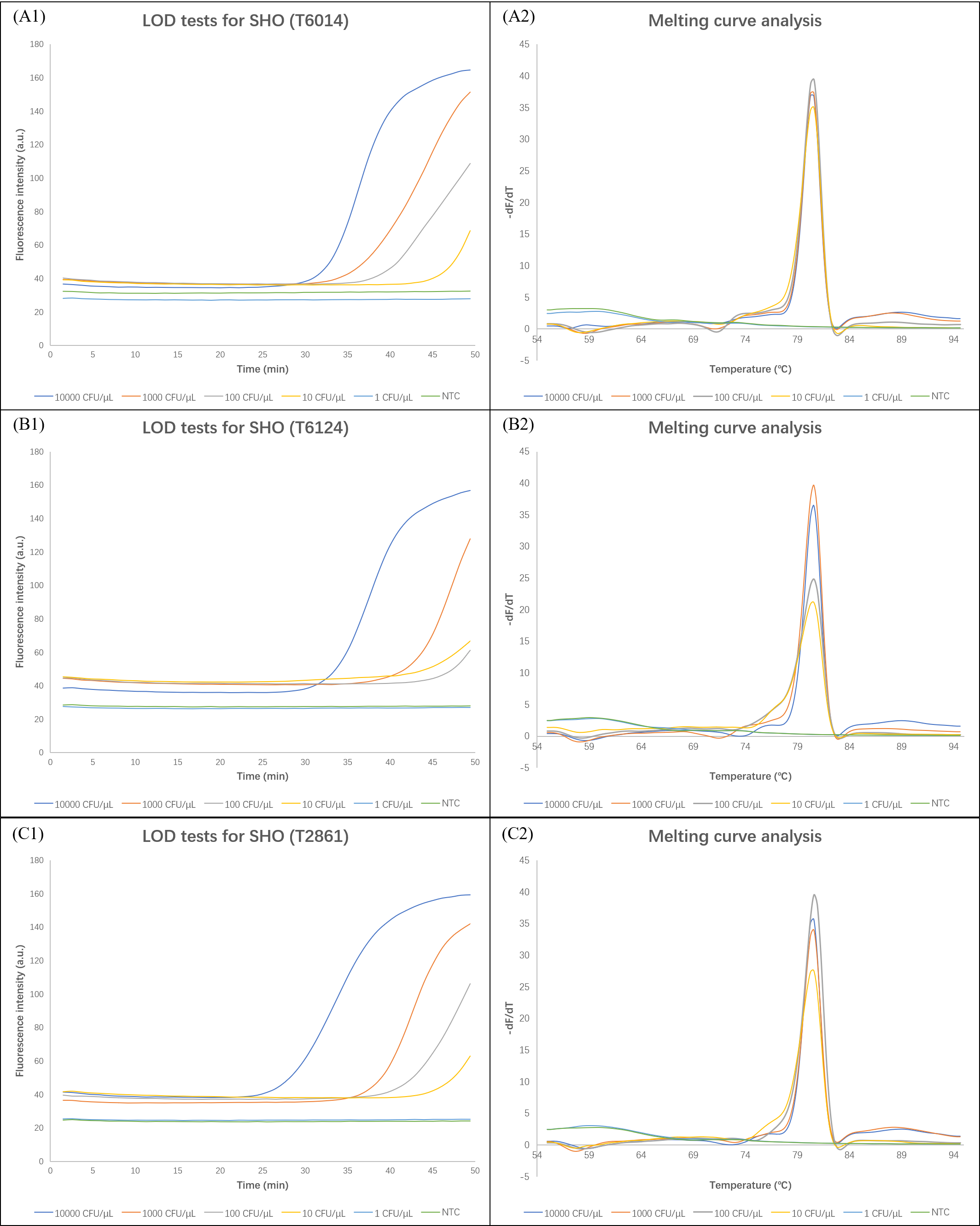

Supplement: Supplementary file 7 [file Image_7.TIF]

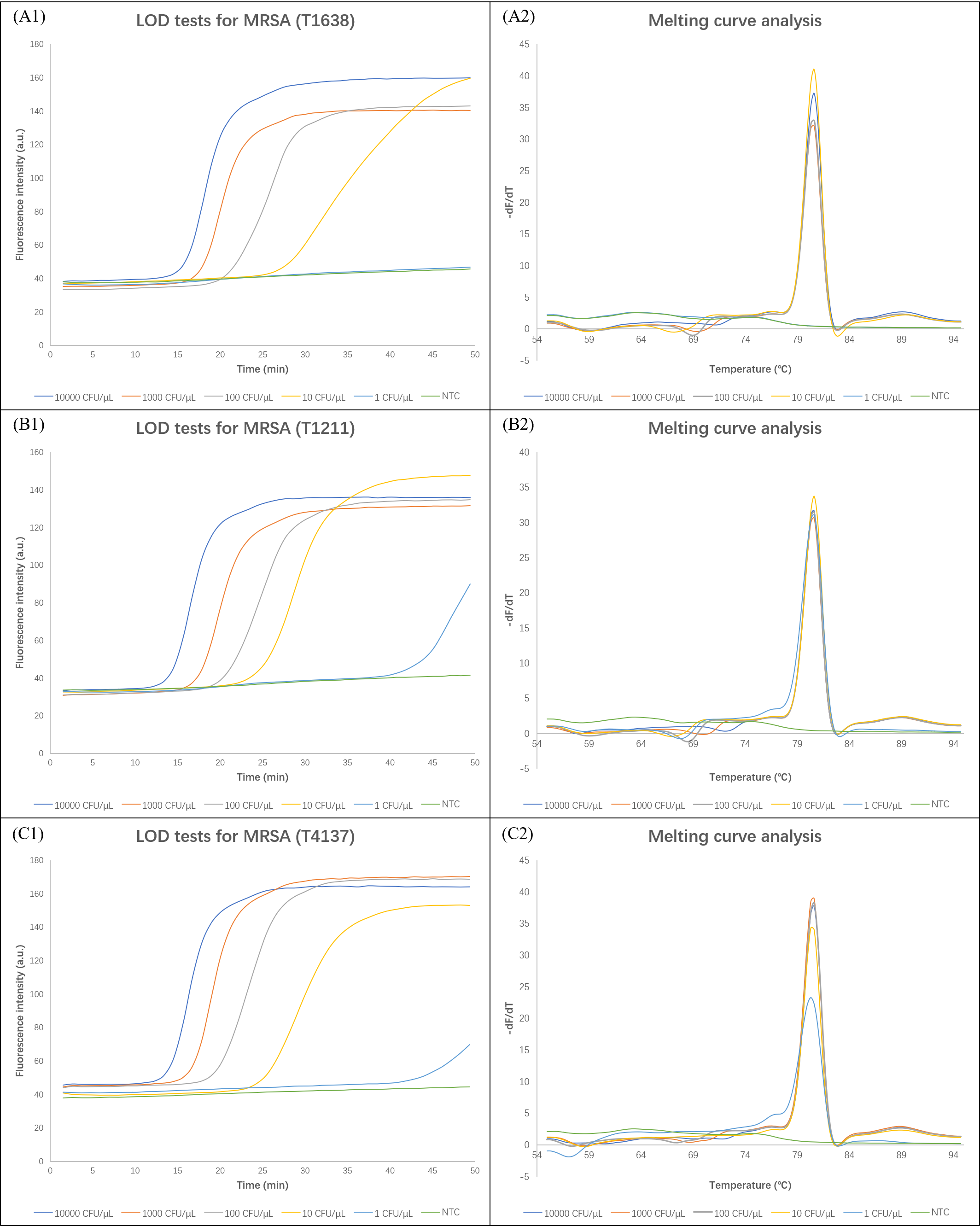

Supplement: Supplementary file 8 [file Image_8.TIF]
