# Supplementary material for: Rapid Detection of mecA and femA Genes by Loop-Mediated Isothermal Amplification in a Microfluidic System for Discrimination of Different Staphylococcal Species and Prediction of Methicillin Resistance
Source: Front Microbiol. 2020 Jul 9;11:1487. doi: 10.3389/fmicb.2020.01487 (PMC7367217; doi:10.3389/fmicb.2020.01487)
Supplement: Supplementary file 9 [file Data_Sheet_1.docx]

**Table S1.** The sequences of PCR primers and probe for *mecA* gene

| **Name** | **Sequence** |
| --- | --- |
| Forward primer | CATATGAGATAGGCATCGTTCCAA |
| Reverse primer | TGCGAAATCWCTTAAATATTCATCCAT |
| Probe | FAM-ATGATACCTTCGTTCCACTTAAAACCGT-TAMRA |

**Table S2.** Substances embedded in each reaction chamber in advance

| **Reaction chamber** | **Substances embedded in advance** |
| --- | --- |
| 1 | Positive control template and primers |
| 2 | —— |
| 3 | Primers for *mecA* gene |
| 4 | Primers for *femA-SAU* gene |
| 5 | Primers for *femA-SEP* gene |
| 6 | Primers for *femA-SHA* gene |
| 7 | Primers for *femA-SHO* gene |
| 8 | —— |
| 9 | —— |
| 10 | —— |

**Table S3.** The sequences of positive control template and primers

| **Name** | **Sequence** |
| --- | --- |
| Positive control template | CTTACTGGTCGCCCATCTCACACTCATGCCTTCCACATTCCACTTAGCGACTAAGTCATTATTACTATGGGGACGGGTTGTTCTTGAACGATGCTATACTTCGTATAGGAAGCCGTTTTTTTATGCCCCATCCTTTCATATGTTCCATAGCACAAGAATGTTCTCTACAGGAGAAGTGCCTATAGGGCTGCAGCTGCAGTTTTGGCCAAGAAATAGAACCAAAGCCAAATTTATT |
| F3 | GCCCATCTCACACTCATG |
| B3 | TGGCCAAAACTGCAGCT |
| FIP | TCAAGAACAACCCGTCCCC-CCTTCCACATTCCACTTAGCG |
| BIP | TAGGAAGCCGTTTTTTTATGCCC-CCTGTAGAGAACATTCTTGTGC |
| LF | ATAGTAATAATGACTTAGT |
| LB | CATCCTTTCATATGTTCCA |

**Table S4.** Repeatability of fluorescent intensity detection

| **Concentration of fluorescent dye/μM** | **Reaction chamber** | **Average** | **SD** | **CV/%** |
| --- | --- | --- | --- | --- |
| 0.1 | 1 | 10461.5 | 43.15 | 0.41 |
|  | 2 | 10798.4 | 46.63 | 0.43 |
|  | 3 | 11331.4 | 43.54 | 0.38 |
|  | 4 | 11358.0 | 40.62 | 0.36 |
|  | 5 | 11532.1 | 30.29 | 0.26 |
|  | 6 | 11515.0 | 75.15 | 0.65 |
|  | 7 | 11093.4 | 46.44 | 0.42 |
|  | 8 | 11264.1 | 21.75 | 0.19 |
|  | 9 | 10989.0 | 48.00 | 0.44 |
|  | 10 | 11307.1 | 75.48 | 0.67 |
| 0.2 | 1 | 15351.2 | 62.34 | 0.41 |
|  | 2 | 16424.5 | 44.81 | 0.27 |
|  | 3 | 16815.0 | 69.83 | 0.42 |
|  | 4 | 17017.7 | 48.03 | 0.28 |
|  | 5 | 17046.2 | 63.87 | 0.37 |
|  | 6 | 17011.6 | 67.51 | 0.40 |
|  | 7 | 17117.6 | 31.58 | 0.18 |
|  | 8 | 16853.6 | 52.22 | 0.31 |
|  | 9 | 16760.2 | 53.03 | 0.32 |
|  | 10 | 16648.0 | 48.12 | 0.29 |
| 0.4 | 1 | 27257.7 | 104.74 | 0.38 |
|  | 2 | 28387.9 | 106.21 | 0.37 |
|  | 3 | 28613.0 | 102.12 | 0.36 |
|  | 4 | 29039.9 | 103.12 | 0.36 |
|  | 5 | 29319.2 | 49.20 | 0.17 |
|  | 6 | 29390.0 | 104.48 | 0.36 |
|  | 7 | 29051.8 | 42.91 | 0.15 |
|  | 8 | 29028.3 | 89.90 | 0.31 |
|  | 9 | 28593.0 | 93.33 | 0.33 |
|  | 10 | 27965.7 | 57.58 | 0.21 |
| 0.6 | 1 | 40201.4 | 62.97 | 0.16 |
|  | 2 | 41964.3 | 54.04 | 0.13 |
|  | 3 | 42679.2 | 102.86 | 0.24 |
|  | 4 | 42934.1 | 97.80 | 0.23 |
|  | 5 | 42901.4 | 136.72 | 0.32 |
|  | 6 | 42982.3 | 175.28 | 0.41 |
|  | 7 | 42714.5 | 126.88 | 0.30 |
|  | 8 | 42867.9 | 175.19 | 0.41 |
|  | 9 | 42334.5 | 165.69 | 0.39 |
|  | 10 | 42059.5 | 99.89 | 0.24 |
| 0.8 | 1 | 55037.2 | 147.82 | 0.27 |
|  | 2 | 57402.1 | 139.68 | 0.24 |
|  | 3 | 58437.2 | 193.96 | 0.33 |
|  | 4 | 58879.7 | 77.96 | 0.13 |
|  | 5 | 59017.1 | 164.01 | 0.28 |
|  | 6 | 59408.7 | 144.34 | 0.24 |
|  | 7 | 58897.8 | 159.36 | 0.27 |
|  | 8 | 58603.8 | 183.93 | 0.31 |
|  | 9 | 57920.5 | 101.89 | 0.18 |
|  | 10 | 56990.8 | 129.39 | 0.23 |

**Table S5.** Precision of fluorescent intensity detection

| **Concentration of fluorescent dye/μM** | **Reaction chamber** | **Fluorescence intensity** | **Average** | **SD** | **CV/%** |
| --- | --- | --- | --- | --- | --- |
| 0.1 | 1 | 10461.5 | 11165.00 | 336.26 | 3.01 |
|  | 2 | 10798.4 |  |  |  |
|  | 3 | 11331.4 |  |  |  |
|  | 4 | 11358.0 |  |  |  |
|  | 5 | 11532.1 |  |  |  |
|  | 6 | 11515.0 |  |  |  |
|  | 7 | 11093.4 |  |  |  |
|  | 8 | 11264.1 |  |  |  |
|  | 9 | 10989.0 |  |  |  |
|  | 10 | 11307.1 |  |  |  |
| 0.2 | 1 | 15351.2 | 16704.56 | 519.65 | 3.11 |
|  | 2 | 16424.5 |  |  |  |
|  | 3 | 16815.0 |  |  |  |
|  | 4 | 17017.7 |  |  |  |
|  | 5 | 17046.2 |  |  |  |
|  | 6 | 17011.6 |  |  |  |
|  | 7 | 17117.6 |  |  |  |
|  | 8 | 16853.6 |  |  |  |
|  | 9 | 16760.2 |  |  |  |
|  | 10 | 16648.0 |  |  |  |
| 0.4 | 1 | 27257.7 | 28664.65 | 660.80 | 2.31 |
|  | 2 | 28387.9 |  |  |  |
|  | 3 | 28613.0 |  |  |  |
|  | 4 | 29039.9 |  |  |  |
|  | 5 | 29319.2 |  |  |  |
|  | 6 | 29390.0 |  |  |  |
|  | 7 | 29051.8 |  |  |  |
|  | 8 | 29028.3 |  |  |  |
|  | 9 | 28593.0 |  |  |  |
|  | 10 | 27965.7 |  |  |  |
| 0.6 | 1 | 40201.4 | 42363.91 | 843.43 | 1.99 |
|  | 2 | 41964.3 |  |  |  |
|  | 3 | 42679.2 |  |  |  |
|  | 4 | 42934.1 |  |  |  |
|  | 5 | 42901.4 |  |  |  |
|  | 6 | 42982.3 |  |  |  |
|  | 7 | 42714.5 |  |  |  |
|  | 8 | 42867.9 |  |  |  |
|  | 9 | 42334.5 |  |  |  |
|  | 10 | 42059.5 |  |  |  |
| 0.8 | 1 | 55037.2 | 58059.49 | 1302.97 | 2.24 |
|  | 2 | 57402.1 |  |  |  |
|  | 3 | 58437.2 |  |  |  |
|  | 4 | 58879.7 |  |  |  |
|  | 5 | 59017.1 |  |  |  |
|  | 6 | 59408.7 |  |  |  |
|  | 7 | 58897.8 |  |  |  |
|  | 8 | 58603.8 |  |  |  |
|  | 9 | 57920.5 |  |  |  |
|  | 10 | 56990.8 |  |  |  |

**Table S6.** Heating rate of the instrument

| **Channel** | **Time/s** | **Temperature/℃** | **Mean heating rate/°C•min^-1^** |
| --- | --- | --- | --- |
| 1 | 346~431 | 37.29~64.54 | 19.23 |
| 2 | 347~434 | 37.49~64.52 | 18.64 |
| 3 | 346~425 | 37.33~64.56 | 20.68 |
| 4 | 344~433 | 37.49~64.51 | 18.20 |

**Table S7.** Cooling rate of the instrument

| **Channel** | **Time/s** | **Temperature/℃** | **Mean cooling rate/°C•min^-1^** |
| --- | --- | --- | --- |
| 1 | 4015~4120 | 64.54~37.41 | 15.50 |
| 2 | 4016~4122 | 64.74~37.44 | 15.45 |
| 3 | 4016~4121 | 64.75~37.44 | 15.61 |
| 4 | 4017~4123 | 64.71~37.40 | 15.46 |

**Table S8** Temperature control accuracy of the instrument

|  | **Time/s** | **Channel 1/℃** | **Time/s** | **Channel 2/℃** | **Time/s** | **Channel 3/℃** | **Time/s** | **Channel 4/℃** |
| --- | --- | --- | --- | --- | --- | --- | --- | --- |
|  | 461 | 64.81 | 464 | 64.81 | 455 | 65.17 | 463 | 64.92 |
|  | 462 | 64.81 | 465 | 64.81 | 456 | 65.17 | 464 | 64.92 |
|  | 463 | 64.80 | 466 | 64.81 | 457 | 65.17 | 465 | 64.92 |
|  | 464 | 64.80 | 467 | 64.81 | 458 | 65.17 | 466 | 64.92 |
|  | 465 | 64.79 | 468 | 64.81 | 459 | 65.17 | 467 | 64.92 |
|  | 466 | 64.79 | 469 | 64.81 | 460 | 65.17 | 468 | 64.92 |
|  | 467 | 64.79 | 470 | 64.81 | 461 | 65.17 | 469 | 64.92 |
|  | 468 | 64.79 | 471 | 64.81 | 462 | 65.17 | 470 | 64.93 |
|  | 469 | 64.78 | 472 | 64.81 | 463 | 65.17 | 471 | 64.93 |
|  | 470 | 64.78 | 473 | 64.80 | 464 | 65.16 | 472 | 64.93 |
|  | 471 | 64.77 | 474 | 64.80 | 465 | 65.16 | 473 | 64.93 |
|  | 472 | 64.77 | 475 | 64.80 | 466 | 65.16 | 474 | 64.93 |
|  | 473 | 64.77 | 476 | 64.80 | 467 | 65.15 | 475 | 64.93 |
|  | 474 | 64.77 | 477 | 64.80 | 468 | 65.15 | 476 | 64.93 |
|  | 475 | 64.76 | 478 | 64.80 | 469 | 65.15 | 477 | 64.92 |
|  | 476 | 64.76 | 479 | 64.80 | 470 | 65.14 | 478 | 64.92 |
|  | 477 | 64.76 | 480 | 64.80 | 471 | 65.14 | 479 | 64.92 |
|  | 478 | 64.75 | 481 | 64.80 | 472 | 65.14 | 480 | 64.92 |
|  | 479 | 64.75 | 482 | 64.80 | 473 | 65.14 | 481 | 64.92 |
|  | 480 | 64.75 | 483 | 64.80 | 474 | 65.14 | 482 | 64.92 |
|  | 481 | 64.75 | 484 | 64.80 | 475 | 65.13 | 483 | 64.92 |
|  | 482 | 64.75 | 485 | 64.79 | 476 | 65.13 | 484 | 64.93 |
|  | 483 | 64.74 | 486 | 64.79 | 477 | 65.12 | 485 | 64.93 |
|  | 484 | 64.74 | 487 | 64.79 | 478 | 65.12 | 486 | 64.93 |
|  | 485 | 64.73 | 488 | 64.79 | 479 | 65.12 | 487 | 64.93 |
|  | 486 | 64.73 | 489 | 64.79 | 480 | 65.12 | 488 | 64.93 |
|  | 487 | 64.72 | 490 | 64.79 | 481 | 65.12 | 489 | 64.93 |
|  | 488 | 64.71 | 491 | 64.79 | 482 | 65.11 | 490 | 64.93 |
|  | 489 | 64.71 | 492 | 64.78 | 483 | 65.11 | 491 | 64.93 |
|  | 490 | 64.71 | 493 | 64.78 | 484 | 65.11 | 492 | 64.93 |
| **Average/℃** |  | 64.76 |  | 64.80 |  | 65.15 |  | 64.93 |
| **Deviation/℃** |  | -0.24 |  | -0.20 |  | 0.15 |  | -0.07 |

**Figure S1.** Schematic illustration of the instrument.

**Figure S2.** Schematic illustration of the whole procedure for staphylococcal identification and methicillin resistance prediction by our method.

**Figure S3.** The linearity of fluorescence intensity with concentration of fluorescent dye.

**Figure S4.** Results of the LOD tests for *S. aureus* using clinically isolated strains. (A1-C1) Amplification curves of the LOD tests for SAU T1638, SAU T1211, SAU T4137, respectively, and (A2-C2) the corresponding melting curve analysis.

(LOD=limit of detection, dF/dT=derivative of the fluorescence/derivative of the temperature, SAU=*Staphylococcus aureus*, NTC= no template control)

**Figure S5.** Results of the LOD tests for *S. epidermidis* using clinically isolated strains. (A1-C1) Amplification curves of the LOD tests for SEP T6749, SEP T6969, SEP T6183, respectively, and (A2-C2) the corresponding melting curve analysis.

(LOD=limit of detection, dF/dT=derivative of the fluorescence/derivative of the temperature, SEP=*Staphylococcus epidermidis*, NTC= no template control)

**Figure S6.** Results of the LOD tests for *S. haemolyticus* using clinically isolated strains. (A1-C1) Amplification curves of the LOD tests for SHA T5975, SHA T7183, SHA T7597, respectively, and (A2-C2) the corresponding melting curve analysis.

(LOD=limit of detection, dF/dT=derivative of the fluorescence/derivative of the temperature, SHA=*Staphylococcus haemolyticus*, NTC= no template control)

**Figure S7.** Results of the LOD tests for *S. hominis* using clinically isolated strains. (A1-C1) Amplification curves of the LOD tests for SHO T6014, SHO T6124, SHO T2861, respectively, and (A2-C2) the corresponding melting curve analysis.

(LOD=limit of detection, dF/dT=derivative of the fluorescence/derivative of the temperature, SHO=*Staphylococcus hominis*, NTC= no template control)

**Figure S8.** Results of the LOD tests for methicillin-resistant *S. aureus* using clinically isolated strains. (A1-C1) Amplification curves of the LOD tests for MRSA T1638, MRSA T1211, MRSA T4137, respectively, and (A2-C2) the corresponding melting curve analysis.

(LOD=limit of detection, dF/dT=derivative of the fluorescence/derivative of the temperature, MRSA=methicillin-resistant *Staphylococcus aureus*, NTC= no template control)
